# Impact of functional and technical quality on patient satisfaction in prosthetic and orthotic care: A cross-sectional study

**DOI:** 10.1371/journal.pone.0333481

**Published:** 2025-10-03

**Authors:** Mahmoud Alfatafta, Nizar Alsubahi, Huda Alfatafta, Huthaifa Atallah, Amneh Alshawabka, Anthony McGarry, Alaeddin Ahmad

**Affiliations:** 1 Department of Prosthetics and Orthotics, School of Rehabilitation Sciences, The University of Jordan, Amman, Jordan; 2 Department of Health Services and Hospitals Administration, Faculty of Economics and Administration, King Abdulaziz University, Jeddah, Saudi Arabia; 3 Department of Health Services Research, Faculty of Health, Medicine and Life Sciences, Care and Public Health Research Institute—CAPHRI, Maastricht University Medical Center, Maastricht University, Maastricht, Maryland, The Netherlands; 4 Faculty of Health Sciences, University of Pecs, Pecs, Hungary; 5 Biomedical Engineering, The University of Strathclyde, Glasgow, United Kingdom; 6 Department of Marketing, School of Business, The University of Jordan, Amman, Jordan; St Mary's University, UNITED STATES OF AMERICA

## Abstract

Patient satisfaction with prosthetic and orthotic services is shaped by both functional quality, such as communication and service accessibility, and technical quality, including device durability and usability. This study examined how these dimensions are associated with patient satisfaction in diverse healthcare settings in Jordan, a low- to middle-income country. A cross-sectional survey of 307 users from government, military, private, and non-governmental (NGO) providers was conducted using a 28-item questionnaire developed from existing literature. Confirmatory factor analysis and structural equation modeling confirmed the scale’s validity and tested associations between variables. Both functional quality (β = 0.521, p < 0.001) and technical quality (β = 0.382, p < 0.001) were significantly associated with satisfaction, with functional quality showing a stronger influence. NGO users reported the highest satisfaction scores, and the model explained 41.7% of the variance in satisfaction. ANOVA results revealed statistically significant differences among provider types. These findings highlight the value of patient-centered care and suggest that enhancing communication, accessibility, and service responsiveness may improve satisfaction, particularly in public and private sectors.

## Introduction

Prosthetic and orthotic (P&O) services play a crucial role in enhancing mobility, functionality, and overall quality of life for individuals with limb loss or musculoskeletal impairments. Globally, millions of people rely on prosthetic and orthotic devices, with estimates suggesting that over 100 million individuals require assistive mobility devices due to limb amputation, congenital limb differences, or neurological conditions [1–3]. Despite advancements in prosthetic and orthotic technologies, disparities in service quality persist across different healthcare settings, particularly in low and middle-income countries (LMICs), where access to high-quality care remains a significant challenge [4].

Patient satisfaction with P&O services is a key indicator of healthcare quality and may influence device adherence, long-term functionality and overall rehabilitation success [5]. Two critical dimensions affecting patient satisfaction are functional quality (FQ) which refers to the patient experience, provider communication, and service accessibility and technical quality (TQ) which encompasses device durability, performance, and usability [5,6]. While both dimensions are essential, recent studies suggest that patient-centered care and functional quality may have a greater influence on satisfaction than technical aspects alone [7]. However, limited research has examined how these two quality components interact in diverse healthcare settings, particularly in countries with fragmented service delivery models.

Jordan serves as a relevant example of a low- to middle-income country (LMIC) with diverse P&O service providers, which include government-funded centers, military hospitals, private clinics, and non-governmental organizations (NGOs) [8–10]. Similar to other LMICs, Jordan’s healthcare system faces challenges in standardizing service quality, with previous reports indicating variations in patient satisfaction across different provider types [9]. Understanding how functional and technical quality impact patient satisfaction in this context provides valuable insights for healthcare policymakers, clinicians, and manufacturers worldwide, particularly in regions facing similar challenges.

Several studies have examined patient satisfaction in prosthetic and orthotic settings, particularly in high-income countries. For example, Ramstrand et al. (2024) conducted a large-scale national study in Sweden and found that patient-centered functional quality dimensions were more predictive of satisfaction than technical features. Similarly, Pezzin et al. (2004) reported that emotional support, accessibility, and follow-up care were essential satisfaction predictors in the U.S. context. A study by DadeMatthews et al. (2024) linked prosthesis satisfaction with both service quality and device functionality in a sample of lower-limb prosthesis users. However, these studies were conducted in highly regulated environments with standardized care pathways and do not account for the variability of service delivery common in low- and middle-income countries [5,11,12].

Limited evidence exists from LMICs where multiple provider types coexist, often leading to inconsistent care standards. For example, Ghoseiri and Bahramian (2012) explored user satisfaction in a single clinic in Iran, while Simadi and Almomani (2008) evaluated patient perspectives toward NGO services in Jordan, but neither study addressed both functional and technical service dimensions nor employed structural equation modeling [13,14].

The current study addresses this gap by assessing how functional quality (FQ) and technical quality (TQ) independently influence satisfaction within a fragmented healthcare system. Structural equation modeling (SEM) was used to evaluate these relationships and to compare satisfaction outcomes across four distinct provider types (government, military, private, and NGO) marking one of the first investigations of its kind in an LMIC context. This research contributes to the global effort to enhance patient-centered P&O care, improve service delivery models, and inform policies that may promote equitable access to high-quality prosthetic and orthotic services.

## Methods

### Design

This study employed a cross-sectional, survey-based methodology to investigate the relationships between patient satisfaction (PS), functional quality (FQ), and technical quality (TQ). The survey instrument was developed following a comprehensive literature review on prosthetic and orthotic service quality and patient satisfaction (Ramstrand et al., 2024; Bettoni et al., 2016; Simadi and Almomani, 2008; Biddiss and Chau, 2007; Pezzin et al., 2004; Faezipour and Ferreira, 2013).

A 28-item questionnaire (S1 Appendix) was designed to assess FQ (16 items), TQ (7 items), and PS (5 items) using a five-point Likert scale ranging from 1 (strongly disagree) to 5 (strongly agree) [15]. The item pool was initially generated by reviewing established instruments in prosthetics, orthotics, and general healthcare settings [5,16–18]. Items were adapted to suit the Jordanian context with attention to language clarity and cultural appropriateness.

Existing validated instruments, such as the Trinity Amputation and Prosthesis Experience Scales (TAPES) and the cross-cultural equivalence testing of the Prosthetic Evaluation Questionnaire (PEQ), were reviewed but found to be unsuitable for the primary objectives of this study. TAPES focuses predominantly on psychosocial adjustment and user experience, while the PEQ is designed for clinical outcome measurement in prosthetic users rather than for evaluating service quality dimensions across different provider types [19,20]. Because this study aim was to capture perceptions of both functional and technical quality in prosthetic and orthotic services within Jordan’s multi-provider context, a tailored questionnaire was developed. Items were adapted from published instruments in prosthetics, orthotics, and healthcare quality literature to ensure conceptual grounding. Content validity was confirmed through expert panel review, and psychometric testing was conducted using confirmatory factor analysis (CFA).

To ensure content validity, the draft questionnaire was reviewed by five domain experts representing prosthetic and orthotic clinicians, health services researchers, and academic faculty. The experts evaluated item clarity, relevance, and comprehensiveness. Minor revisions were incorporated based on their feedback. The final version underwent psychometric testing using confirmatory factor analysis (CFA).

Before the main study, the questionnaire was piloted with a small group of prosthetic and orthotic users (n = 10) to ensure clarity and cultural appropriateness. Feedback from the pilot highlighted minor wording adjustments for two items (“delivery time” and “clarity of communication”), which were revised prior to full-scale distribution (see S2 Appendix).

A priori sample size considerations were based on recommendations for structural equation modeling (SEM), which suggest a minimum of 10 participants per estimated parameter to ensure model stability and reliable estimation [21]. With 28 observed items loading onto three latent constructs, the minimum recommended sample size was approximately 280 participants. The final sample of 307 exceeded this threshold, providing sufficient statistical power (estimated at > 0.90) to detect medium effect sizes (β ≥ 0.30) at α = 0.05 (see S3 Appendix). This sample also allowed for meaningful subgroup comparisons, although the private provider subgroup (n = 11) was recognized as underpowered for some analyses and is noted as a limitation of the study.

The study adhered to STROBE guidelines for observational studies (EQUATOR Network). The survey was carefully designed to gather relevant information directly from research respondents, ensuring that their responses reflect first hand insights and opinions. Ethical approval for this study was provided by the Jordanian Ministry of Health, National Ethics Committee for Health Research (MOH/REC/2023/415). All procedures involving human participants were conducted in accordance with the ethical standards of the institutional and national research committees and with the 1964 Declaration of Helsinki and its later amendments or comparable ethical standards.

### Participants

Participants were eligible if they had received prosthetic or orthotic services from one of the four primary provider types in Jordan (Royal Medical Services (Military sector, 1 center), Ministry of Health (1 center), Private sector (7 centers) and NGOs (3 centers). Posters were pinned onto notice boards at each provider as well as study adverts that were available as a handout in the waiting areas.

Respondents were required to be 12 years or older to participate in the study. For participants under 18 years old, written parental or guardian consent was obtained in accordance with the ethical guidelines of the National Ethics Committee for Health Research. All participants provided written informed consent before completing the questionnaire. Participants were also required to have the ability to understand the questions and communicate verbally without any cognitive impairment. Participation was entirely voluntary, and surveys were administered and collected on the same day to ensure consistency in data collection. To mitigate risks associated with social desirability and recall bias inherent in self-reported data, the survey was administered anonymously, and no identifying information was collected. Participants were informed that their responses would be kept confidential and used solely for research purposes. The survey items were phrased neutrally to avoid leading responses and to promote honest reporting. Research assistants were trained to provide clarification without influencing participant answers, and the survey was completed in a single sitting to reduce recall distortion. The recruitment period for this study started on 1^st^ January 2024 and ended on 2^nd^ July 2024. All participants aged 18 and over provided written informed consent, and for those under 18, written parental or guardian consent was obtained prior to participation.

### Data analysis

Data were analyzed using a multi-step statistical approach to ensure rigor and robustness. Statistical analyses were conducted using SPSS (version 29) and AMOS (version 28) software.

Descriptive statistics were employed to summarize the demographic characteristics of participants, including age, gender, and service provider. Frequencies, percentages, means, and standard deviations were calculated for all variables to provide an overview of the study population.

Normality of the data was assessed using Shapiro–Wilk tests, histograms, and Q-Q plots, which confirmed that the dataset met the assumptions of normal distribution required for subsequent analyses (see S4 Appendix).

Cronbach’s alpha (α) was employed as a reliable indicator for evaluating internal consistency. Values exceeding the 0.70 threshold were deemed acceptable [22]. Convergent validity was assessed using composite reliability (CR) and average variance extracted (AVE). A CR > 0.70 and AVE > 0.50 were considered indicative of sufficient convergent validity [21,23]. Discriminant validity was evaluated by comparing the square root of the AVE for each construct with its correlations with other constructs, ensuring that AVE values were greater than any inter-construct correlation coefficients [23]. Conformity factor analysis (CFA) was conducted to validate the measurement model and confirm the constructs’ dimensionality. Factor loadings were examined to ensure all items met the minimum loading threshold of 0.50 [21]. Items with loadings below this threshold were excluded. Following the confirmatory factor analysis (CFA), model fit indices were calculated to evaluate how well the measurement model aligned with the observed data. The indices indicated an acceptable fit, with all values meeting or approaching recommended thresholds [24] (e.g., χ²/df between 1–3, CFI ≥ 0.90, TLI ≥ 0.90, NFI ≥ 0.90, IFI ≥ 0.90, and RMSEA < 0.08). S5 Appendix presents the detailed model fit statistics, demonstrating that the measurement model provided a satisfactory representation of the underlying constructs.

Structural Equation Modelling (SEM) was used to test the hypothesized relationships between functional quality (FQ), technical quality (TQ), and patient satisfaction (PS). Path coefficients (β), standard errors (S.E.), critical ratios (C.R.), and significance levels (p-values) were reported. The R-squared (R^2^) value was calculated to determine the variance in patient satisfaction explained by FQ and TQ.

ANOVA tests were conducted to explore differences in FQ, TQ, and PS across the four service provider groups (Government, Military, Private, NGOs). Post-hoc tests (Tukey’s HSD) were performed to identify pairwise differences where significant main effects were observed.

ANOVA tests were conducted to examine the relationship between service provider as a demographic data element and research variables separately for FQ, TQ and PS. This analysis helped to determine whether there were significant differences in the research variables across each service provider.

Although subgroup sample sizes varied, especially in the private provider group (n = 11), statistical assumptions such as homogeneity of variance were reviewed, and post-hoc tests were interpreted cautiously considering these limitations.

## Results

### Demographic data

A total of 400 research questionnaires were distributed to all the patients in the four main providers. Of the 400 questionnaires, 337 questionnaires were retrieved, and 30 were excluded due to their lack of validity for statistical analysis. Thus, 307 were analyzed (76.7%). Participants’ details are presented in Table 1.

**Table 1:** Socio-demographic characteristics of the respondents (N = 307).

Item		Frequency	%
Age	18 and less	80	26.1
	19-29	46	15.0
	30-49	102	33.2
	50 and More	79	25.7
Sex	Female	115	37.5
	Male	192	62.5
Service Provider	Government (MOH)	160	52.1
	Military	94	30.6
	Private	11	3.6
	NGOs	42	13.7
	**Total**	**307**	**100.0**

Demographic data revealed that the highest number of respondents 33.2% were between 30–49 years old. Male respondents comprised approximately 63 percent of all completed surveys. Lastly, a total of 52.1% of the respondents were from Government (MOH) centers. In terms of device type, 234 participants were prosthetic users, while 73 were orthotic users.

### Validity and reliability by Confirmatory Factor Analysis (CFA)

Table 2 illustrates the mean values and standard deviation for each of the independent variables (FQ and TQ) and dependent variable (PS). Mean scores ranged from 3.726 to 4.208. The highest-rated variable was PS (Mean = 4.208, SD = 0.593) and the lowest was FQ (Mean = 3.726, SD = 0.636) meanwhile the TQ was (Mean = 4.098, SD = 0.618). In the same way, Table 2 presents item loadings between 0.700 and 0.830, surpassing the recommended threshold of 0.50 or higher [21]. Convergent validity, assessed through composite reliability (CR) and average variance extracted (AVE), indicated strong internal consistency with CR values ranging from 0.842 to 0.902 and AVE values from 0.581 to 0.596, exceeding the 0.7 and 0.50 benchmarks, respectively [23]. All latent variables meet the convergent validity standard. Additionally, Cronbach alpha values for all constructs exceed 0.70, ranging from 0.838 to 0.899, affirming their reliability. Furthermore, the discriminant validity refers to the extent to which factors are distinct and uncorrelated, the results are presented in Table 2. Table 2 shows the results of the AVE analysis. It can be seen that the AVE values are above 0.5 and, moreover, are greater than any correlation coefficients between constructs.

**Table 2:** Reliability analysis and factor analysis results.

*Latent Variable*	*Indicator*	*FL*	*FLS*	AVE (> 0.50)	CR (> 0.70)	Cronbach’s Alpha
Functional Quality (FQ) Mean = 3.726 SD = 0.636 Discriminant Validity Assessment = **0.767**	F1 welcome received	0.770	0.593	0.589	0.902	0.899
	F2 waiting time	0.800	0.640			
	F3 level of privacy	0.760	0.578			
	F4 conference with medical doctor	0.750	0.563			
	F5 guidance provided	0.740	0.548			
	F6 attention and time allocated for questions	0.720	0.518			
	F7 delivery time	0.710	0.504			
	F8 contacts with health insurance	0.700	0.490			
	F9 patient first	0.830	0.689			
	F10 reachable by telephone	0.770	0.593			
	F11 reachable by public transport	0.790	0.624			
	F12 parking spaces	0.790	0.624			
	F13 waiting-rooms	0.820	0.672			
	F14 clarity of the communication	0.810	0.656			
	F15 patient concerns	0.750	0.563			
	F16 adequate information	0.760	0.578			
Technical Quality (TQ) Mean = 4.098 SD = 0.618 Discriminant Validity Assessment = **0.772**	T1 information provided	0.820	0.672	0.596	0.842	0.838
	T2 cosmetics of device	0.800	0.640			
	T3 easy to use	0.790	0.624			
	T4 durable device	0.730	0.533			
	T5 skin abrasion and irritation device	0.740	0.548			
	T6 pain free	0.760	0.578			
	T7 clear instructions	0.760	0.578			
Patient Satisfaction (PS) Mean = 4.208 SD = 0.593 Discriminant Validity Assessment = **0.762**	PS1 accessibility	0.760	0.578	0.581	0.868	0.860
	PS2 provided device	0.740	0.548			
	PS3 consultations	0.800	0.640			
	PS4 revisit	0.780	0.608			
	PS5 continuity and movement	0.730	0.533			

FL = Factor Loading, FLS = Factor Loading Squared, AVE = Average Variance Extracted, CR = Composite Reliability.

Functional quality had a significant impact on patient satisfaction at P = .000, Beta = 0.521. Similarly, technical quality had a significant impact on patient satisfaction at P = .000, Beta = 0.382. The value of R-Square of patient satisfaction was 0.417 (combined R^2^), which means that 41.7% of patient satisfaction was explained by functional and technical quality (Table 3).

**Table 3:** Structural equation modelling regression weights.

			Estimate	S.E.	C.R.	P	Effect	R	R^2^
**FQ**	→	**PS**	0.521	0.031	25.275	***	0.521	0.521^**^	0.271
**TQ**	→	**PS**	0.382	0.037	21.607	***	0.382	0.382^**^	0.145

S.E. = Standard errors of the regression weights, C.R. = Critical Ratio, P = p-value (* < 0.05, ** < 0.01, *** < 0.001)

### Path analysis of (SEM)

ANOVA results indicated that the highest satisfaction ratings were observed among NGO providers, with a mean PS score of 4.56, followed by military (4.31), private (4.33), and government services (4.05). Similarly, NGOs scored the highest in both TQ (4.47) and FQ (4.30), while government-run services had the lowest scores in both dimensions (TQ = 3.95, FQ = 3.41) see (S6 Appendix).

## Discussion

This study demonstrates that both functional quality (FQ) and technical quality (TQ) significantly influence patient satisfaction among prosthetic and orthotic users, with functional quality exerting a stronger effect. Findings revealed that FQ (β = 0.521, p < 0.001) had a greater impact on patient satisfaction than TQ (β = 0.382, p < 0.001), explaining 42.6% of the variance in satisfaction. Service providers affiliated with NGOs received the highest patient satisfaction ratings, while government-run centers consistently scored the lowest, indicating disparities in service quality across different healthcare sectors. These results highlight the critical importance of patient-centered care and effective provider-patient interactions in enhancing user satisfaction with prosthetic and orthotic services (Tables 2 and 3).

Unlike other studies such as Ramstrand et al. (2024), which was conducted in a highly standardized Swedish system [5], this study examined how service quality dimensions function in a fragmented LMIC context. This distinction provides novel insights into the interplay between provider type, functional quality, and patient satisfaction in settings where services are not harmonized.

The findings of this study (Tables 2, 3) align with global healthcare trends, where functional aspects of care, such as communication, responsiveness, and patient-provider relationships, often outweigh purely technical considerations in influencing satisfaction [11]. Recent studies confirm that patient-centered care models, which prioritize accessibility, interaction quality, and emotional support, lead to higher satisfaction and better adherence to assistive devices [11].

While technical quality is still significant, its reduced influence suggests that patients may be more forgiving of certain technical imperfections if the overall care experience is positive (Table 3). This aligns with previous findings [11], where patient satisfaction was closely linked to how well the device integrated into daily activities, regardless of technical sophistication. Similar to this study findings, Pezzin et al. (2004) reported that patient satisfaction with prosthetic services was influenced by multiple factors, including technical performance (75.7% satisfaction) as well as functional outcomes such as mobility and comfort. Their study highlights that while technical aspects remain critical, patients ultimately judge device success by how well it supports their daily activities and functional needs [12].

The results revealed significant differences in patients’ satisfaction based on the type of service provider. Lower satisfaction scores associated with government-run P&O services may reflect systemic limitations, such as resource constraints and high patient volumes, which hinder the delivery of individualized care. These findings are consistent with studies in other LMIC contexts where public prosthetic and orthotic facilities faced challenges in staffing, user follow-up, and responsiveness to patient needs [13,14]. This underscores the importance of service delivery structure in shaping user experience within prosthetic and orthotic care models.

In contrast, NGO providers, which focus on patient engagement and tailored services, consistently achieve higher satisfaction ratings, supporting previous studies indicating that personalized service models foster greater patient trust and adherence to prosthetic and orthotic interventions [13]. Literature shows similar results [14]. These results also showed that NGOs excel in patient-centered care, providing robust guidance and clear communication, which likely explains their strong patient satisfaction scores.

NGOs consistently outperformed other providers in patient satisfaction, a finding that may be attributed to several unique structural and operational factors. NGOs often operate with a strong emphasis on personalized, patient-centered care, supported by flexible service delivery models that are less constrained by bureaucratic or institutional hierarchies [25]. NGOs may have greater autonomy in allocating resources, hiring qualified staff, and tailoring services to individual patient needs. NGO services in Jordan often receive support from international organizations, enabling access to more advanced prosthetic components and materials without the cost restrictions commonly found in public facilities [9]. This external funding and technical support may also allow NGOs to implement higher training standards, continuous professional development, and outreach programs that build trust and rapport with the communities they serve. Future research should investigate these practices in depth to determine which structural or behavioral interventions contribute most to patient satisfaction.

Interestingly, military healthcare services scored higher than private providers in technical quality, suggesting that military users receive more durable and high-functionality devices, a trend also noted in research on prosthetic rehabilitation among veterans and physically demanding occupations [11]. This underscores the need for wider civilian access to advanced prosthetic solutions, particularly for highly active individuals.

While this study explains 42.6% of the variance in patient satisfaction, other important factors such as psychosocial elements, economic considerations, and demographic influences may also play a role in shaping satisfaction [26–28].

The findings have significant implications for healthcare policy, service delivery, and future research in prosthetic and orthotic care, both within Jordan and in the broader global healthcare context. Policymakers should prioritize improving patient-provider communication and accessibility in government-run prosthetic and orthotic centers, ensuring that patients receive adequate guidance on device use and personalized care. National healthcare frameworks should incorporate minimum standards for patient-centered care to guarantee that prosthetic and orthotic users, regardless of the service provider, receive comparable levels of support. Greater investment in partnerships with NGOs may help bridge gaps in service quality, particularly in resource-limited public facilities, a challenge that extends beyond Jordan to many low- and middle-income countries facing similar disparities in healthcare access.

From a clinical perspective, healthcare providers should focus on training personnel in communication skills and patient-centered care approaches to enhance patient satisfaction. The findings suggest that patients value usability and practical guidance on device use over purely technical advancements, reinforcing the need to integrate structured education and rehabilitation training into prosthetic and orthotic services. Military healthcare providers, who often emphasize high-functionality prosthetic designs for physically demanding roles, should consider adopting these advanced solutions for broader civilian applications, particularly for active individuals in need of durable and high-performance devices.

Based on the study’s findings, improving functional quality, particularly communication and accessibility, should be a priority for government and private providers. Strategies may include staff training in patient-centered communication, the use of clear informational materials in Arabic, and implementation of standardized consultation protocols. To improve accessibility, clinics could reduce waiting times through appointment systems, extend service hours, and ensure reachability via public transport or targeted subsidies. Enhancing continuity of care through patient follow-up systems (e.g., SMS reminders) and improving waiting room environments can also contribute to a more positive experience. Furthermore, offering emotional support and integrating routine patient feedback into service design can help align care more closely with user needs. These practices, successfully adopted by NGOs, represent actionable steps that other sectors can adapt to improve patient satisfaction and overall care quality.

Future research should explore longitudinal outcomes to assess how functional and technical quality impact long-term device use, patient adherence, and device abandonment rates, as these are critical factors influencing healthcare efficiency and resource allocation. Given the rise of advanced prosthetic technologies such as neural integration and AI-driven myoelectric prostheses, further investigation is needed to understand how these innovations balance functional and technical attributes in shaping patient satisfaction, usability, and long-term health outcomes. Additionally, research on regional disparities in prosthetic and orthotic care should extend beyond Jordan to examine underserved populations in other low-resource settings, where access to high-quality prosthetic care remains a pressing challenge. Understanding these broader global healthcare trends will help inform future policies and drive improvements in service provision, ensuring equitable access to high-quality prosthetic and orthotic care worldwide.

A major strength of this study is its use of structural equation modeling (SEM) and confirmatory factor analysis (CFA), which provided robust validation of the relationships between FQ, TQ, and patient satisfaction. The inclusion of a diverse sample from multiple service providers (government, military, private, and NGO) enhances the generalizability of findings. Additionally, this study contributes to global discussions on healthcare quality by reinforcing the importance of functional service attributes over technical aspects, aligning with broader trends in patient-centered healthcare [11]. The study’s findings are relevant to other healthcare systems, particularly in low- and middle-income countries (LMICs), where disparities in prosthetic and orthotic services remain a challenge [4].

This study is subject to several limitations. First, its cross-sectional design limits the ability to draw causal inferences about the relationships between service quality dimensions and patient satisfaction. Longitudinal research is recommended to capture changes in perceptions over time and across different rehabilitation stages. Second, the study relies on self-reported data, which introduces the potential for recall bias and social desirability effects. Although anonymity and neutral question phrasing were used to reduce these risks, future studies should incorporate objective measures or triangulate findings using clinician-reported or behavioral data. Third, prosthetic and orthotic users were analyzed as a single group due to the integrated nature of service delivery in Jordan, where both populations are commonly treated within the same facilities by the same clinical teams. This reflects real-world practice in the local context. However, the Authors acknowledge that prosthesis and orthosis users have different functional dependencies, clinical goals, and expectations. Grouping them may mask important subgroup-specific satisfaction patterns. Future research should consider stratified analyses based on device type (e.g., upper vs. lower limb, prosthetic vs. orthotic) to provide more targeted insights into user experiences and service quality dimensions.

Although demographic characteristics such as age and gender were reported descriptively, the analysis did not stratify satisfaction outcomes by age group. It is possible that younger users (e.g., adolescents or young adults) may have different satisfaction thresholds or expectations compared to older adults, especially given differences in life roles, rehabilitation goals, and adaptation periods. Future studies should consider age-stratified analyses or include age as a moderating variable in modeling approaches. In addition, other sociodemographic data such as income level, education, employment status, and disability classification were collected. These factors were excluded from the present analysis to maintain analytical focus but may provide deeper insight into satisfaction patterns. Future studies should incorporate these variables to identify potential disparities and refine recommendations across different patient subpopulations. Finally, while concerns about provider overlap across settings have been noted in other countries, this is not applicable in Jordan, where strict regulations prohibit clinicians from working across multiple healthcare sectors (e.g., public, military, private, NGO) simultaneously. While the total sample size (N = 307) was adequate for structural equation modeling and overall model validation, subgroup comparisons, particularly involving the private sector (n = 11), should be interpreted with caution. The small sample size in this subgroup may reduce statistical power, affect variance assumptions in ANOVA, and limit the generalizability of findings for private sector users. Future research should aim for more balanced subgroup sizes to allow for more robust between-group comparisons.

## Conclusion

This study confirms that both functional and technical quality significantly impact patient satisfaction among prosthetic and orthotic users, with functional quality encompassing patient interactions, service convenience, and communication having a stronger effect. Higher satisfaction with NGO services underscores the value of user-centered care, while lower satisfaction in other sectors highlights areas for improvement. These findings align with prior research emphasizing the link between service quality and patient outcomes. Enhancing both technical aspects and patient-provider interactions can improve satisfaction, reduce device abandonment, and promote better long-term outcomes. Future research should focus on interventions that optimize both service dimensions.

## Supporting information

S1 Appendix 28-item questionnaire used to assess functional quality (FQ), technical quality (TQ), and patient satisfaction (PS). (DOCX)

S2 Appendix Pilot testing summary of the 28-item questionnaire. (DOCX)

S3 Appendix Power analysis summary. (DOCX)

S4 Appendix Data normality testing results. (DOCX)

S5 Appendix Model fit statistics. (DOCX)

S6 Appendix ANOVA descriptive statistics table showing mean scores (TQ, FQ, and PS) for each provider type (Government, Military, Private, NGO). (DOCX)
