# Supplementary material for: Impact of functional and technical quality on patient satisfaction in prosthetic and orthotic care: A cross-sectional study
Source: PLoS One. 2025 Oct 3;20(10):e0333481. doi: 10.1371/journal.pone.0333481 (PMC12494285; doi:10.1371/journal.pone.0333481)
Supplement: S1 Appendix — (DOCX) [file pone.0333481.s001.docx]

| Demographic questions | Age |
| --- | --- |
|  | Sex |
|  | Service Provider |
| FQ1 | The welcome received at the prosthetic and orthotic care provider center? |
| FQ2 | The waiting time until your next appointment at the prosthetic and orthotic care provider center? |
| FQ3 | The level of privacy in the fitting rooms at the prosthetic and orthotic care provider center? |
| FQ4 | The conference with medical doctor at the prosthetic and orthotic care provider center? |
| FQ5 | The guidance provided on the usage and maintenance of your device at the prosthetic and orthotic care provider center? |
| FQ6 | The level the attention and time allocated for questions and remarks at the prosthetic and orthotic care provider center? |
| FQ7 | The delivery time for the device at the prosthetic and orthotic care provider center? |
| FQ8 | The contacts with health insurance at the prosthetic and orthotic care provider center |
| FQ9 | The prosthetic and orthotic care provider center puts my interests first? |
| FQ10 | The prosthetic and orthotic care provider center is reachable by telephone? |
| FQ11 | The prosthetic and orthotic care provider center is reachable by means of public transport? |
| FQ12 | The parking spaces at the prosthetic and orthotic care provider center? |
| FQ13 | The waiting-rooms at the prosthetic and orthotic care provider center? |
| FQ14 | How would you rate the clarity of the communication you received from your Prosthetic and orthotic care provider center? |
| FQ15 | Did your Prosthetic and orthotic care provider center listen to your concerns and answer your questions? |
| FQ16 | Were you provided with adequate information about your device, including their purpose and potential side effects? |
| TQ1 | The information provided about the device at the prosthetic and orthotic care provider center? |
| TQ2 | Your wishes regarding the cosmetics of your device have been met at the prosthetic and orthotic care provider center? |
| TQ3 | The prosthetic and orthotic care provider center gives me easy to put on prosthetics or orthotics. |
| TQ4 | The prosthetic and orthotic care provider center gives me durable prosthetics or orthotics. |
| TQ5 | The prosthetic and orthotic care provider center gives me Skin abrasion and irritation prosthetics or orthotics. |
| TQ6 | The prosthetic and orthotic care provider center me pain free prosthetics or orthotics. |
| TQ7 | Were you provided with clear instructions on how to manage your device at home? |
| PS1 | I am satisfied with accessing Prosthetic and orthotic care provider center services |
| PS2 | I am satisfied with the provided device. |
| PS3 | I am satisfied with the consultations provided by the Prosthetic and orthotic care provider center staff. |
| PS4 | I will revisit the Prosthetic and orthotic care provider center if I experience any other problem with device. |
| PS5 | I am satisfied with the continuity and movement within the Prosthetic and orthotic care provider center departments. |

S1 Appendix. 28-item questionnaire used to assess functional quality (FQ), technical quality (TQ), and patient satisfaction (PS).
