# Supplementary material for: Impact of functional and technical quality on patient satisfaction in prosthetic and orthotic care: A cross-sectional study
Source: PLoS One. 2025 Oct 3;20(10):e0333481. doi: 10.1371/journal.pone.0333481 (PMC12494285; doi:10.1371/journal.pone.0333481)
Supplement: S2 Appendix — (DOCX) [file pone.0333481.s002.docx]

S2 Appendix. Pilot testing Summary of the 28-item questionnaire

The pilot aimed to evaluate the clarity, cultural relevance, and practicality of the 28-item questionnaire before full implementation.

**Methods**

Ten prosthetic and orthotic users (6 prosthesis users, 4 orthosis users; aged 19–62 years) were recruited from one NGO clinic. Participants completed the draft questionnaire and provided feedback on item clarity and comprehension.

**Results**

**Overall completion time:** Average 12 minutes (range: 9–15 minutes).

**Clarity feedback:** 26 of 28 items were rated clear by all participants.

**Revisions made:** Minor wording changes were made to “delivery time” (Item 7) and “clarity of communication” (Item 14).

**No items** were flagged as culturally inappropriate or irrelevant.

Table S1. Pilot test participant profile and feedback summary (n=10)

| Device type | Age | Clarity issues reported | Time to complete (min) |
| --- | --- | --- | --- |
| Prosthesis | 45 | None | 11 |
| Orthosis | 34 | None | 10 |
| Prosthesis | 58 | “Delivery time” wording | 13 |
| Prosthesis | 21 | None | 9 |
| Orthosis | 29 | None | 12 |
| Prosthesis | 62 | “Clarity of communication” phrasing | 15 |
| Prosthesis | 37 | None | 10 |
| Orthosis | 19 | None | 11 |
| Prosthesis | 50 | None | 12 |
| Orthosis | 40 | None | 13 |

**Conclusion**

The pilot confirmed the questionnaire was clear, culturally relevant, and practical, requiring only minor wording adjustments before being finalized for the main study.
