# Supplementary material for: Impact of functional and technical quality on patient satisfaction in prosthetic and orthotic care: A cross-sectional study
Source: PLoS One. 2025 Oct 3;20(10):e0333481. doi: 10.1371/journal.pone.0333481 (PMC12494285; doi:10.1371/journal.pone.0333481)
Supplement: S3 Appendix — (DOCX) [file pone.0333481.s003.docx]

| **Analysis type** | **Effect size (f²)** | **α Error probability** | **Power (1-β)** | **Required sample size** | **Actual sample size** | **Achieved power** |
| --- | --- | --- | --- | --- | --- | --- |
| SEM Model (FQ + TQ → PS) | 0.15 (medium) | 0.05 | 0.80 | 92 | 307 | 0.99 |
| ANOVA (4 provider groups) | 0.25 (medium) | 0.05 | 0.80 | 180 | 307 | 0.95 |
| CFA (28 items, 3 constructs) | N/A | 0.05 | 0.80 | 200 | 307 | 0.98 |

S3 Appendix Power analysis summary. Note: Post hoc power analysis was conducted using GPower 3.1 to confirm that the achieved sample size (N = 307) exceeded the minimum required for SEM, CFA, and ANOVA tests.
