# Supplementary material for: Impact of functional and technical quality on patient satisfaction in prosthetic and orthotic care: A cross-sectional study
Source: PLoS One. 2025 Oct 3;20(10):e0333481. doi: 10.1371/journal.pone.0333481 (PMC12494285; doi:10.1371/journal.pone.0333481)
Supplement: S4 Appendix — (DOCX) [file pone.0333481.s004.docx]

| **Descriptive Statistics** | | | | | | |
| --- | --- | --- | --- | --- | --- | --- |
|  | N | Mean | Skewness | | Kurtosis | |
|  | Statistic | Statistic | Statistic | Std. Error | Statistic | Std. Error |
| F1 | 307 | 3.98 | -.609 | .139 | -.286 | .277 |
| F2 | 307 | 3.72 | -.594 | .139 | -.222 | .277 |
| F3 | 307 | 3.88 | -.596 | .139 | -.418 | .277 |
| F4 | 307 | 4.05 | -.660 | .139 | -.163 | .277 |
| F5 | 307 | 3.92 | -.527 | .139 | -.295 | .277 |
| F6 | 307 | 3.99 | -.665 | .139 | -.113 | .277 |
| F7 | 307 | 3.71 | -.616 | .139 | -.007 | .277 |
| F8 | 307 | 3.67 | -.679 | .139 | -.035 | .277 |
| F9 | 307 | 3.98 | -.546 | .139 | -.421 | .277 |
| F10 | 307 | 3.87 | -.863 | .139 | -.057 | .277 |
| F11 | 307 | 2.74 | .263 | .139 | -1.056 | .277 |
| F12 | 307 | 2.33 | .559 | .139 | -1.143 | .277 |
| F13 | 307 | 3.36 | -.106 | .139 | -.696 | .277 |
| T1 | 307 | 3.93 | -.580 | .139 | -.101 | .277 |
| T2 | 307 | 3.90 | -.432 | .139 | -.501 | .277 |
| T3 | 307 | 4.27 | -1.249 | .139 | 1.610 | .277 |
| T4 | 307 | 4.27 | -1.247 | .139 | 2.278 | .277 |
| T5 | 307 | 4.08 | -1.113 | .139 | 1.120 | .277 |
| T6 | 307 | 4.04 | -.757 | .139 | .028 | .277 |
| T7 | 307 | 4.19 | -.879 | .139 | 2.366 | .277 |
| PS1 | 307 | 4.11 | -1.031 | .139 | 2.891 | .277 |
| PS2 | 307 | 4.17 | -1.341 | .139 | 3.304 | .277 |
| PS3 | 307 | 4.16 | -1.047 | .139 | 2.032 | .277 |
| PS4 | 307 | 4.32 | -1.946 | .139 | 6.202 | .277 |
| PE6 | 307 | 4.18 | -1.314 | .139 | 2.704 | .277 |
| PS5 | 307 | 4.28 | -1.396 | .139 | 4.571 | .277 |
| Valid N (listwise) | 307 |  |  |  |  |  |

S4 Appendix. Data normality testing results.
