# Supplementary material for: Impact of functional and technical quality on patient satisfaction in prosthetic and orthotic care: A cross-sectional study
Source: PLoS One. 2025 Oct 3;20(10):e0333481. doi: 10.1371/journal.pone.0333481 (PMC12494285; doi:10.1371/journal.pone.0333481)
Supplement: S5 Appendix — (DOCX) [file pone.0333481.s005.docx]

| **χ2** | **χ2/df** | **CFI** | **TLI** | **NFI** | **IFI** | **RMSEA** |
| --- | --- | --- | --- | --- | --- | --- |
| **548.154** | 2.674 | 0.904 | 0.906 | 0.902 | 0.905 | 0.074 |

S5 Appendix. Model fit statistics.
