# Supplementary material for: Impact of functional and technical quality on patient satisfaction in prosthetic and orthotic care: A cross-sectional study
Source: PLoS One. 2025 Oct 3;20(10):e0333481. doi: 10.1371/journal.pone.0333481 (PMC12494285; doi:10.1371/journal.pone.0333481)
Supplement: S6 Appendix — (DOCX) [file pone.0333481.s006.docx]

| **Report** | | | | |
| --- | --- | --- | --- | --- |
| Service Provider | | TQ | FQ | PS |
| Government | Mean | 3.9464 | 3.4066 | 4.0500 |
|  | N | 160 | 160 | 160 |
|  | Std. Deviation | .57874 | .52155 | .55264 |
| Military | Mean | 4.2021 | 3.9940 | 4.3085 |
|  | N | 94 | 94 | 94 |
|  | Std. Deviation | .53959 | .49401 | .52357 |
| Private | Mean | 4.0000 | 3.8977 | 4.3273 |
|  | N | 11 | 11 | 11 |
|  | Std. Deviation | 1.02817 | .89586 | .63418 |
| NGO | Mean | 4.4694 | 4.2991 | 4.5571 |
|  | N | 42 | 42 | 42 |
|  | Std. Deviation | .61367 | .55808 | .68934 |
| Total | Mean | 4.0982 | 3.7262 | 4.2085 |
|  | N | 307 | 307 | 307 |
|  | Std. Deviation | .61818 | .63640 | .59348 |

The distribution based on service provider has a significant influence on all variables.

| **ANOVA Test** | | | | | | |
| --- | --- | --- | --- | --- | --- | --- |
|  | | Sum of Squares | df | Mean Square | F | Sig. |
| TQ | Between Groups | 10.594 | 3 | 3.531 | 10.061 | .000 |
|  | Within Groups | 106.345 | 303 | .351 |  |  |
|  | Total | 116.938 | 306 |  |  |  |
| FQ | Between Groups | 37.190 | 3 | 12.397 | 43.304 | .000 |
|  | Within Groups | 86.741 | 303 | .286 |  |  |
|  | Total | 123.931 | 306 |  |  |  |
| PS | Between Groups | 10.220 | 3 | 3.407 | 10.581 | .000 |
|  | Within Groups | 97.558 | 303 | .322 |  |  |
|  | Total | 107.778 | 306 |  |  |  |

**S6 Appendix.** ANOVA descriptive statistics table showing mean scores (TQ, FQ, and PS) for each provider type (Government, Military, Private, NGO).
